# Renal denervation, adjusted drugs, or combined therapy for resistant hypertension: A meta-regression

**DOI:** 10.1097/MD.0000000000003939

**Published:** 2016-07-29

**Authors:** Xiao-Yu Qi, Bin Cheng, Ying-Li Li, Yue-Feng Wang

**Affiliations:** a Department of Internal Medicine-Cardiovascular, Daqing Oilfield General Hospital, Daqing, China; b Department of Pharmacy, Daqing Oilfield General Hospital, Daqing, China; c School of Nursing, Daqing Campus Harbin Medical University, Daqing, China.

**Keywords:** adjusted drugs, combined therapy, meta-analysis, RD, resistant hypertension

## Abstract

The objective of this study is to systematically evaluate the efficacy of renal denervation (RD), adjusted drugs, or combined therapy for resistant hypertension (RH) through a systematic review and meta-analysis of controlled studies.

Publications were comprehensively searched. Studies that investigated the effects of RD and/or adjusted drugs in lowering blood pressure (BP) were included. After quality assessment and data extraction, subgroup analyzes were first performed according to blinding method. Meta-regression and inverted funnel plots were also conducted.

A total of 13 studies containing 1604 RH patients were included. Compared with control, the meta-analysis showed that RD significantly reduced office-based BP and ambulatory BP in 6 months in the unblinded studies, while no significant difference was found in the blinded studies. Meta-regression demonstrated the significant influence of blinding method on BP reduction, and further analysis revealed a significant BP reduction compared with baseline even in the control arm of blinded studies. RD had similar effects compared with adjusted drugs, and combined therapy seemed to further reduce the level of BP.

The efficacy of RD was different between blinded and unblinded studies, and our data revealed a significant BP-lowering effect in the control arm of blinded studies, which was helpful to explain this finding. Furthermore, RD seemed to be equivalent to adjusted drugs, and also we suggested a potential advantage of combined therapy of RD and adjusted drugs compared with monotherapy for RH. However, more studies are warranted to better address the issue.

## Abstract 2

Supplemental Digital Content is available in the text

## 1 Introduction

The reported prevalence of hypertension in the globe was 26.4% of adults with a total number of 972 million, and it was estimated that the prevalence would be 29.2% with 1.56 billion patients in 2025.^[1]^ Among them, the proportion of resistant hypertension (RH) accounted for about 10% to 20%,^[2]^ which indicated that the patients need to adopt at least 3 kinds of antihypertensive drugs including a diuretic agent, regardless of controlled blood pressure (BP) or not (this type was also called true RH).^[3]^ Although RH did not account for the majority of hypertension, more and more attentions were focused on it in clinic. As true RH was always associated with many other risk factors, such as increased age, being overweight, diabetes, smoking, elevated creatinine, sleep apnea, and previous cardiovascular disease,^[3,4]^ the risk of severe cardiovascular events increased undoubtedly together with long-standing and high level of BP.

Sympathectomy acted as a radical and surgical procedure for severe hypertensive patients without available drugs previously.^[5]^ Due to its high invasion and disability, as well as the development of antihypertensive drugs, the clinical application was gradually abandoned. Recently, a catheter-based renal denervation (RD) system was designed to ablate sympathetic fibers along renal artery, and this option was attempted to treat RH through inhibiting sympathetic outflow to renal by radiofrequency energy.^[6]^ Meta-analysis based on noncontrolled studies had reported the primary clinical outcomes of RD, and found both promising BP-lowering effects and rarely related complications.^[2,7]^ Meanwhile, Europe and Canada approved the application of RD, and guidelines also mentioned that such a technique may be considered for drug-ineffective patients. A series of controlled studies and randomized controlled studies (RCTs) adopted kinds of outcome measures, including ambulatory BP, office-based BP, and home-based BP, and were further designed to investigate the BP-lowering effect.^[8–22]^ The efficacy of RD, adjusted drugs, and combined therapy of both RD and adjusted drugs compared with monotherapy of adjusted drugs were all covered.

However, the results of BP-lowering effect seemed to be varied across the studies. A new published article revealed the significance of blinding method to BP-lowering effect, and concluded that “adding a randomized control arm does not reduce bias unless it is blinded.”^[23]^ Based on the finding, we performed a meta-regression to further clarify the BP-lowering effect according to blinding method, and also reviewed current evidence of adjusted drugs and the combined therapy.

## 2 Methods

### 2.1 Literature search and study inclusion

To identify all relevant studies investigating the efficacy and safety of RD, adjusted drugs, and combined therapy for RH, online searches were conducted in MEDLINE, EMBASE, the Cochrane Library, and other supplementary sources, such as clinical register center and Google scholar. Search time was up to March 3, 2016. We used search terms as follows: *(renal OR kidney) AND (vascular OR nervous OR nerve) AND denervation AND hypertension*. References, citations and related articles were also screened to increase the recall ratio. Reviews, animal studies, and case series were excluded.

Inclusion of studies was mainly on the bias of screening and reading the titles, abstracts, and full-texts. First, only prospective controlled studies and RCTs were considered for the analysis. Noncontrolled and cross-over studies that compared the BP changes from the level of baseline to post-RD were excluded. Participants were RH patients, who were diagnosed by investigators and physicians. Patients were divided into RD group or control group. The interventions were previous drugs administration (unblinded studies), or sham procedure + previous drugs (blinded studies) in control group, while RD + previous drugs in RD group. The doses and kinds of drugs were stated to be not allowed to alter in each study, except a pharmacist demonstrated a certain BP increase or decrease. Outcome measures should include BP changes and RD-related complications.

### 2.2 Data extraction

After final inclusion, detailed information in each article was extracted to present general characteristic, outcomes data, and methodological quality. General characteristic recorded included first author, publication year, case number, sex, age, intervention, and important factors, such as study design, blinding method, baseline systolic blood pressure (SBP), and diastolic blood pressure (DBP).

### 2.3 Outcome measures

Primary outcome measures were office-based and ambulatory SBP/DBP changes in 3 and 6 months. Secondary outcome measures were nonresponse rate and operation-related complications. Nonresponse was defined as an office-based SBP reduction ≦10 mm Hg in 6 months.

### 2.4 Quality assessment

Quality of RCTs was assessed by the Cochrane tool of risk bias in 7 items: random sequence, allocation concealment, blinding of participant, blinding of outcome assessment, incomplete outcome data, selective reporting result, and other bias. According to the reported information in each study, all the items were judged to be with low, high, or unclear risk of bias. Quality of controlled studies was assessed by the Newcastle–Ottawa Scale recommended by the Cochrane Collaboration in 8 items with a total of 9 stars, and a study achieved more than 5 stars was considered high quality.^[24]^

### 2.5 Data synthesis and analysis

To pooled analyze the data of outcome measures, RevMan (version 5.3, the Cochrane Collaboration, Denmark) was used. To pooled analyze the data of outcomes in single RD arm and control arm, MetaXL (version 4.01, Queensland, Australia) was used. Subgroup analyses were first performed according to the blinding method. We presented overall effects by using mean difference (MD) with respective 95% confidence intervals (95% CI) for continuous data. Statistical heterogeneity was tested by *χ*^2^ statistic and presented by using *I*^*2*^ value. When the value of *I*^*2*^ <50%, the heterogeneity was considered to be nonsignificant, and a fixed-effects model was used. Otherwise, the heterogeneity would be significant, and a random-effects model was used. *P* value <0.05 was judged statistically significant.

### 2.6 Meta-regression and funnel plot

To investigate the source of heterogeneity, and the influence of important factors including study design, blinding method, baseline BP, body mass index, and pulse pressure, meta-regression based on individual study was quantitatively compared. MetaAnlyst (the Agency for Healthcare Research and Quality, United States) was used in random-effects models. Factors were converted to continuous variances, and all the factors were first analyzed by single-factor analysis (significance was set as *P* <0.1), then they were combined analyzed after excluding factors having overlapping effects. Inverted funnel plots were used to assess the risk of publication bias in each outcome measure.

Both of study inclusion and data extraction were completed by 2 independent investigators. The paper was improved by the Preferred Reporting Items for Systematic Reviews and Meta-Analyses (PRISMA) guideline, and did not involve any ethical issue.

## 3 Results

### 3.1 Summary of the included studies

**Figure 1:**
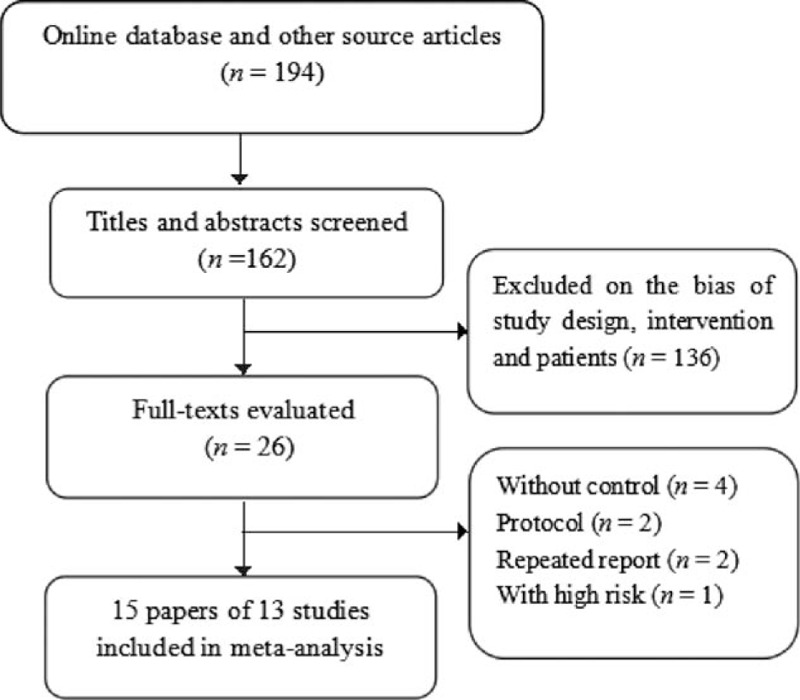
Flow chart of study selection.

A total of 194 publications were initially searched, and finally 15 articles that reported 13 studies containing 1065 patients in RD group and 539 patients in control group were included. The process of study inclusion is shown in Fig. 1, and the general characteristics are shown in Table 1. There were 7 RCTs and 6 controlled studies, and case number ranged from 9 to 341 patients across the studies. Ten studies compared RD with control,^[8–13,15–19,21]^ 2 studies compared RD with adjusted drugs,^[14,20]^ and 1 study compared combined therapy with monotherapy of adjusted drugs.^[22]^ Baseline SBP ranged from 144 to 181 mm Hg and DBP ranged from 81 to 97 mm Hg in RD group. Two kinds of catheters were reported, and 13 studies adopted a Symplicity Cather System except for 1 study adopted a EnglisHTN.^[19]^

**Table 1:**
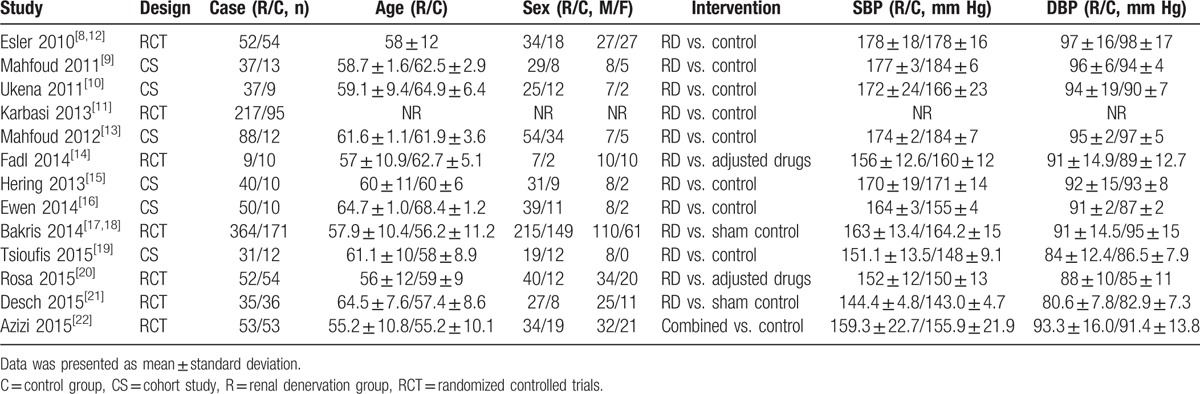
Baseline characteristics of the included trials.

**Figure 2:**
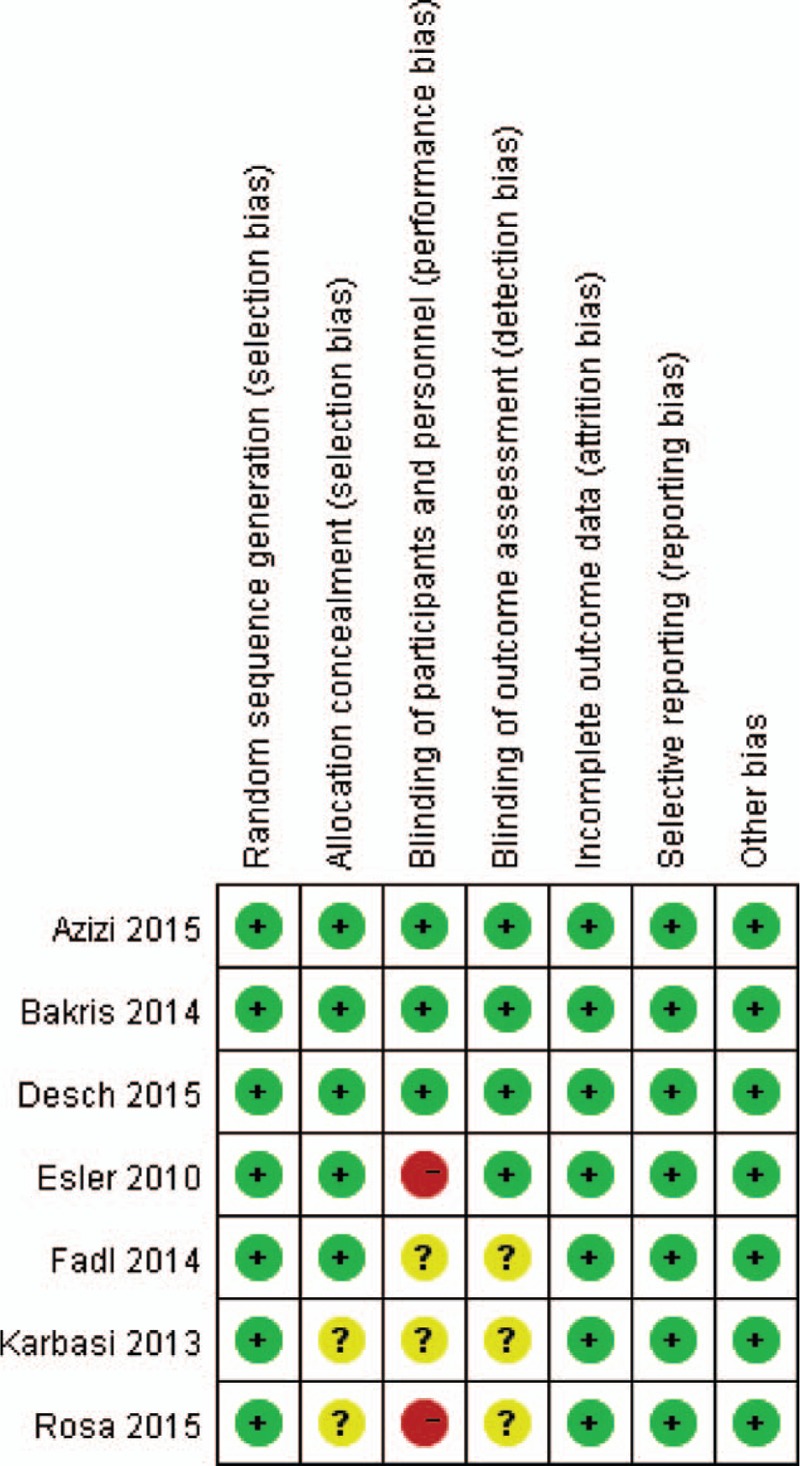
Quality assessment of randomized controlled trials. +, low risk; -, high risk; ?, unclear risk.

Quality assessment of RCTs showed that 2 studies had unclear risk of bias in allocation concealment.^[11,20]^ Only 3 studies had low risk of bias in blinding of participants,^[17,21,22]^ and 4 studies had low risk of bias in blinding of outcome assessment, as shown in Fig. 2. For controlled studies, all of them achieved a total stars ≧ 7, as shown in Table 2.

**Table 2:**
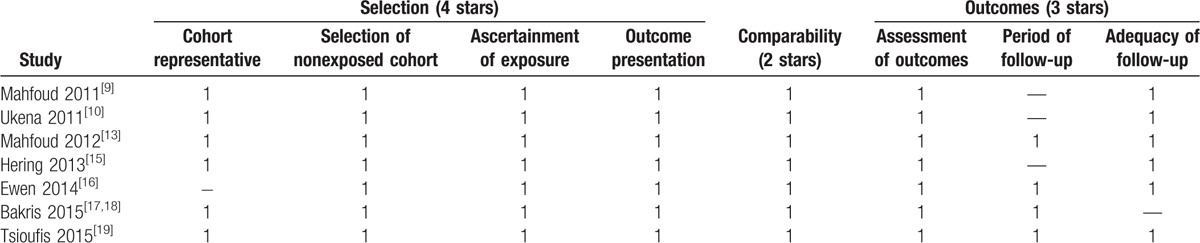
Quality evaluation of included controlled studies.

### 3.2 The effect of RD versus control

#### 3.2.1 Office-based BP in 3 and 6 months

Four unblinded studies reported the data of office-based BP reduction in 3 months. Meta-analysis in random-effects model showed that RD reduced SBP by a mean of 22.92 (95% CI, 13.26–30.59) mm Hg and DBP by a mean of 6.87 (3.41–10.33) mm Hg compared with control.

**Figure 3:**
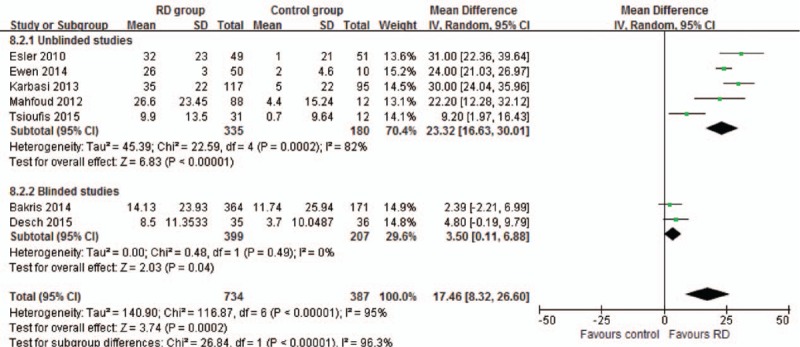
Meta-analysis of systolic blood pressure reduction in 6 months.

**Figure 4:**
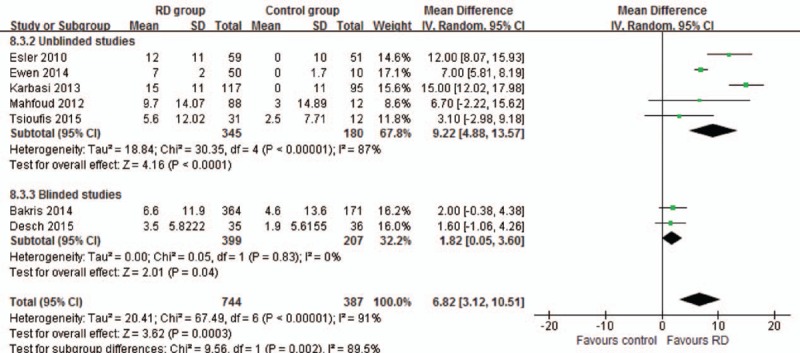
Meta-analysis of diastolic blood pressure reduction in 6 months.

Seven studies reported the data of office-based BP reduction in 6 months. Meta-analysis in random-effects model showed that RD reduced SBP by a mean of 23.32 (16.63–30.01) mm Hg in the unblinded subgroup, while by a mean of 3.5 (0.11–6.88) mm Hg in the blinded subgroup (Fig. 3); RD reduced DBP by a mean of 9.22 (4.88–13.57) mm Hg in the unblinded subgroup, while by a mean of 1.82 (0.05–3.60) mm Hg in the blinded subgroup (Fig. 4).

#### 3.2.2 24 hours ABP in 6 months

Four studies reported the data of 24 hours ABP in 6 months. Meta-analysis in fixed-effects model showed that RD significantly reduced the SBP [MD = 10.97, 95% CI (5.42, 16.52)] and DBP [MD = 5.59, 95% CI (1.19, 9.98)] in the unblinded subgroup. While no significant difference was found in both SBP [MD = 2.2, 95% CI (−0.37, 4.78)] and DBP [MD = 0.90, 95% CI (−0.57, 2.37)] in the blinded subgroup, as shown in Supp Figs. 1 and 2.

### 3.3 Meta-regression

**Figure 5:**
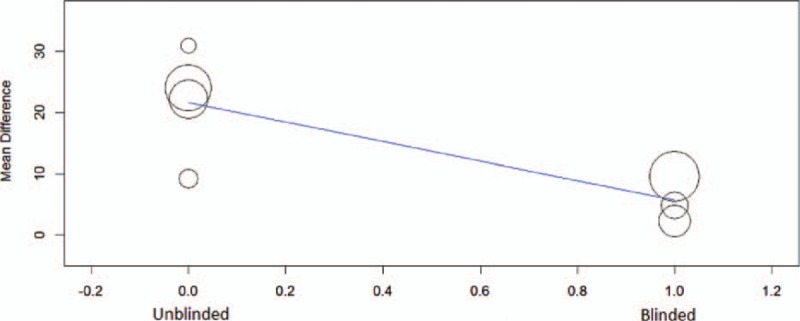
Meta-regression of blinding methods with systolic blood pressure reduction.

To explore the influence of other clinical factors, meta-regression was performed based on the data of office-based SBP reduction in 6 months. Single-factor analysis showed that blinding method (coefficient = −14.18, *P* <0.001, Fig. 5), baseline SBP (coefficient = 0.51, *P* = 0.03), and DBP (coefficient = 0.96, *P* = 0.073) were significant. Further analysis showed that only blinding method was significant in a combined factors analysis [coefficient = −12.45, 95% CI (−21.76, −3.17)].

### 3.4 The influence of blinding method in RD and control arm

#### 3.4.1 Nonresponse rate in only RD arm

**Figure 6:**
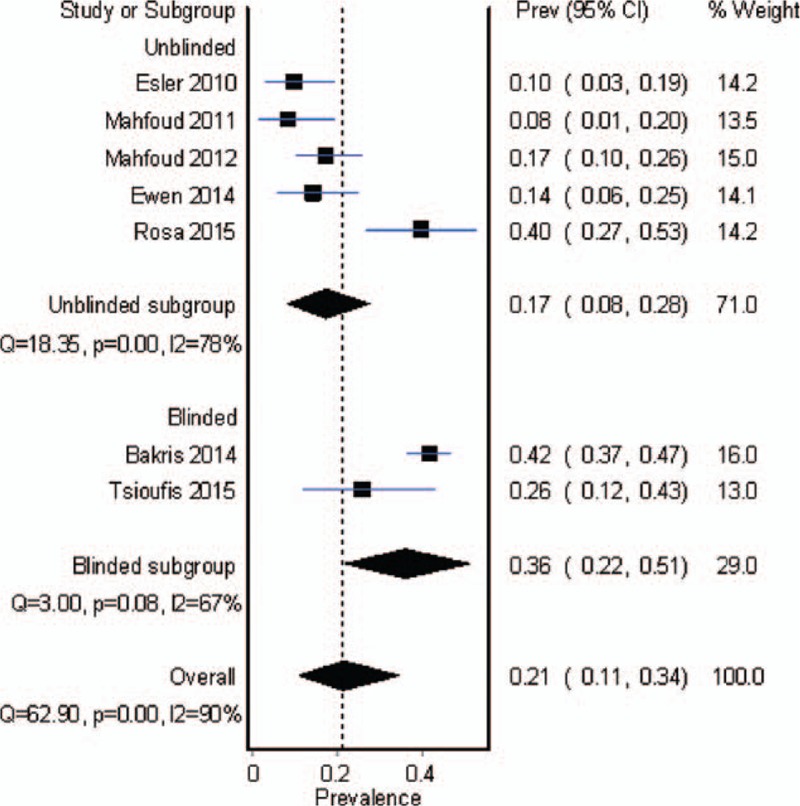
Meta-analysis of nonresponse rate in only renal denervation arm.

Seven studies reported nonresponse rate in RD arm. Meta-analysis in random-effects model showed that the average rate was 17% in unblinded studies and 36% in blinded studies, with an overall rate of 21% (11%–34%), as shown in Fig. 6.

#### 3.4.2 BP changes of baseline in only RD arm

Compared with baseline, both unblinded subgroup and blinded subgroup showed a reduction of office-based SBP and DBP in 6 months. The results of ABP were similar that both unblinded and blinded subgroups showed a significant reduction.

#### 3.4.3 BP changes of baseline in only control arm

**Figure 7:**
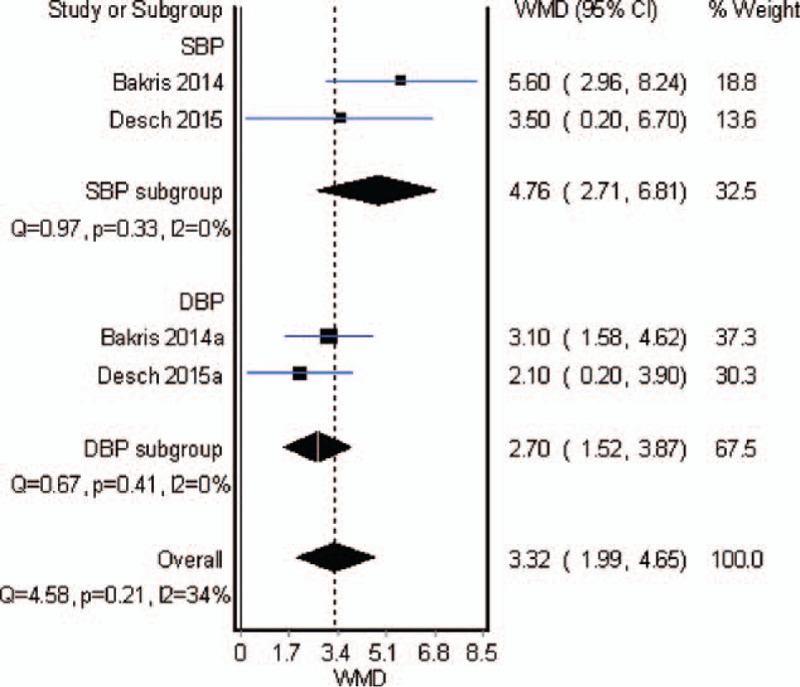
Meta-analysis of systolic blood pressure reduction in control arm of blinded studies in 6 months.

In the subgroup of unblinded studies, both office-based SBP and DBP in 6 months were not significantly changed compared with baseline. But, in the subgroup of blinded studies which additionally adopted a sham-procedure compared with unblinded studies, both office-based SBP [MD = 11.74, 95% CI (7.82, 15.66)] and DBP [MD = 4.60, 95% CI (2.55, 6.65)] were significantly reduced, as shown in Supp Figs. 3 and 4. Meta-analysis of ABP also showed similar trends that ABP did not significantly reduce in the subgroup of unblinded studies in control arm, but in the subgroup of blinded studies, as shown in Fig. 7.

### 3.5 The effect of RD versus adjusted drugs

Two studies compared the results of RD with adjusted drugs in 6 months, and both of them were RCTs. One study included 19 cases,^[14]^ and no significant difference was found in ambulatory SBP (10 ± 12 vs. 19 ± 12, *P* >0.05), although adjusted drugs showed a superiority of office-based SBP reduction (8 ± 15 vs. 28 ± 13, *P* = 0.008). The other study included 106 cases,^[20]^ and reported nonsignificant differences in SBP reduction of both ambulatory [MD = −0.5, 95% CI (−6.1, 5.2)] and office-based [MD = 1.9, 95% CI (−5.2, 9.0)] outcomes.

### 3.6 The effect of combined therapy

Only 1 study containing 101 cases compared combined therapy of RD and adjusted drugs with monotherapy of adjusted drugs. The result showed that combined therapy further achieved a significant reduction of ambulatory SBP [MD = 5.9, 95% CI (0.8, 11.0)], while no difference in office-based SBP [MD = 5.6, 95% CI (−2.0, 13.3)].

## 4 Complications

The included studies compared the indexes of renal function, including eGFR, serum creatinine, and cystatin C, and all of them demonstrated no significant difference. It was reported that RD-related complications were minor and mild during periprocedure, such as femoral artery pseudoaneurysm, transient intraprocedural bradycardia, transient blood pressure drop, and pain.

## 5 Discussion

Our study that was an updated meta-analysis included the latest 7 RCTs and 6 controlled studies. With high reliability, we confirmed the BP-lowering effect of RD. We adopted multiple outcomes to clarify the influence of blinding method to the efficacy of RD for RH, and to be the first reported the estimated nonresponse rate of RD, which was as high as 21%. The efficacy of RD with adjusted drugs, and a potential advantage of combined therapy were also addressed.

To confirm the BP-lowering effect, this study analyzed ambulatory and office-based BP. The combined effect of unblinded studies showed that RD reduced the office-based BP by a mean of 22.92/6.87 mm Hg (SBP/DBP) in 3 months and 23.32/9.22 mm Hg in 6 months, which were similar to previously published studies.^[2,25]^ Also, other studies reported similar reductions in 12 and 24 months.^[26,27]^ However, some differences emerged when the results were limited to only blinded studies. Meta-analysis results showed that RD reduced the office-based BP by a mean of 3.5/1.82 mm Hg in 6 months, which was significantly lower than the level in unblinded studies.

There was no doubt that average ABP was the most certain and reliable index to reflect the real effect of RD. We further analyzed the data of ABP, and the results of unblinded studies still showed a significant reduction of ambulatory BP by a mean of 10.97/5.59 mm Hg in 6 months. But subgroup of blinded studies showed absolutely different results that no statistical difference was found compared with control although RD reduced the ambulatory BP by a mean of 2.2/0.9 mm Hg. Therefore, blinding method influenced the BP-lowering effect of RD for RH.

Current analysis included the latest published papers, among them there were 2 RCTs of Symplicity HTN-3 and FLEX,^[17,21]^ which were designed as large sample, blinded, and sham-controlled trials. However, both of them failed to meet the primary efficacy endpoints. Their combined nonresponse rate was 36%, which was obviously higher than the rate of unblinded studies (17%). Analysis of HTN-3 trials supposed that baseline SBP >180 mm Hg and non-African-American might be the predictors of significant BP reduction.^[28]^ However, available data based on individual study in current meta-regression excluded other potential factors including baseline BP, pulse pressure, and body mass index, while demonstrating the significance of blinding method.

To clarify the influence of blinding method on BP reduction, we first investigated the BP changes compared with baseline in separate RD and control arm. The results confirmed that office-based BP as well as ABP significantly reduced in RD arm regardless of blinding. Besides, the results directly revealed an interesting finding that both ABP and office-based BP significantly reduced also in the control arm of blinded studies. Certainly, the 2 blinded RCTs enabled the reliability of outcomes through enhancing blinding to reduce the risks of performance bias, detection bias (e.g. investigator bias) as well as selection bias.^[29]^ To design such blinded studies of surgical interventions, an additional sham procedure was decisive, and the blinding to all of participants, doctors, and investigators can be realized only when a sham-procedure was first conducted. Thus, the above results supposed that such a sham-procedure seemed to also have “BP-lowering effect.” It was clear that the difference between unblinded and blinded subgroups was mainly caused by the unexpected significant BP reduction in the control arm of blinded studies. However, it was hard to explain why blinded studies showed a significant BP reduction, as they stated that administrated antihypertensive drugs were comparable without statistical differences, and also no reporting of BP-lowering effect of angiography (the sham-procedure) was achieved in databases.

Currently, it was safe that RD was related to minor and mild complications. And it was still recommended as a supplementary treatment on the basis of drugs. As mentioned in the studies, the dose and kinds of antihypertensive agents were mostly not allowed to change, while this intervention seemed actually not to be clinically practical and useful, because BP fluctuation happened commonly in clinic and severe fluctuation must be handled in time. So it was important to evaluate the efficacy of RD compared with adjusted drugs in accordance with clinic, and the results found no significant difference of ABP control in 6 months. And interestingly, what if adjusted drugs combined with RD for RH? However, only 1 study addressed the issue, and concluded that RD further enhanced the effect of antihypertensive drugs.^[22]^

Although RD was designed to ablate the sympathetic nerves around renal artery, and to reduce the activity of sympathetic nerves in overactive patients through inhibiting renin angiotensin aldosterone system without renal function impairment.^[30,31]^ While it seemed that other mechanisms may also be involved when both nerves and vascular were ablated and mechanical dilated. Some studies reported that RD also improved blood glucose and insulin sensitivity,^[9,32]^ and RD even improved augmentation index (which was independent of BP and muscle sympathetic nerve activity) and had beneficial effects on arterial stiffness.^[15]^ Without clear explanations, we suggested that the combined effects of dilation and ablation on vascular and reduced activities of both renal and systemic sympathetic nerves would have complex influences on the whole body.

Limitations and implications for future study were as follows. Different study design would induce a heterogeneity, and especially blinded RCTs eliminated patient’ subjective bias, although suggestive therapy was sometimes specially adopted in clinic. Some studies concerned that potential conflicting of interests might exist for RD device,^[2,33]^ and only 1 study used a different one in our analysis. Another study^[34]^ first reported a PVI catheter, and it was applicable and useful in undeveloped and developing countries. However, our study did not include it as its significant shortcomings in the trial design. Information about experience and learning curve of the doctors performing RD was absent, and the determination and judgement of ablation efficacy were also ignored. Renal noradrenaline spillover or vascular muscle sympathetic nerve activity was reported to be validated to determine the inhibition effect of sympathetic activity,^[6,15]^ while few studies tested them. The study of Mahfoud et al included part of patients in the study of HTN-2,^[9,12]^ and slight overlapping effects may exist. Results of funnel plots suggested risks of publication bias existed (as shown in Supp Fig. 5), which may have negative influence on the outcomes. Besides, more and more studies summarized predictors to ensure the efficacy of RD for specific and true RH patients.^[35]^ All of baseline BP, race, and drug agents were potential predictors, which need to be confirmed. And further BP reduction would be beneficial to reach the treatment goal and to reduce the risk of cardiovascular incidence,^[36]^ although the combined therapy seemed promising, more supports were needed, however.

## 6 Conclusions

The efficacy of RD was different between blinded and unblinded studies, and our data revealed a significant BP-lowering effect in the control arm of blinded studies with a sham procedure, which was helpful to explain this finding. Furthermore, RD seemed to be equivalent to adjusted, and also we suggested potential advantages of combined therapy of RD and adjusted drugs compared with monotherapy for RH. However, more studies are warranted to better address the issue.

## Supplementary Material

Supplemental Digital Content
