# Supplementary material for: Renal denervation, adjusted drugs, or combined therapy for resistant hypertension: A meta-regression
Source: Medicine (Baltimore). 2016 Jul 29;95(30):e3939. doi: 10.1097/MD.0000000000003939 (PMC5265808; doi:10.1097/MD.0000000000003939)

**Supplementary Figures**

***Supp Fig.1*** Meta-analysis of ambulatory systolic blood pressure reduction in 6 months


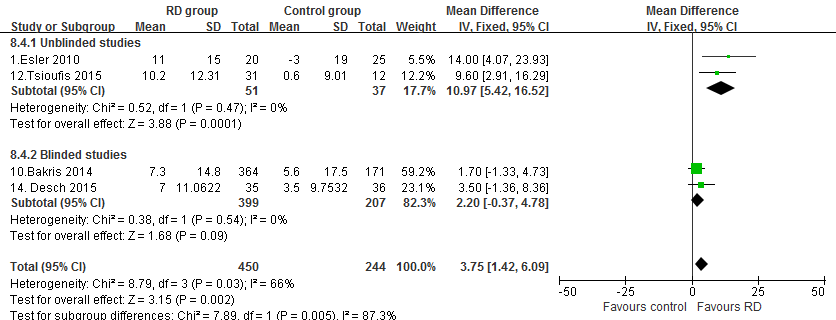


***Supp Fig.2*** Meta-analysis of ambulatory *diastolic blood pressure* reduction in 6 months


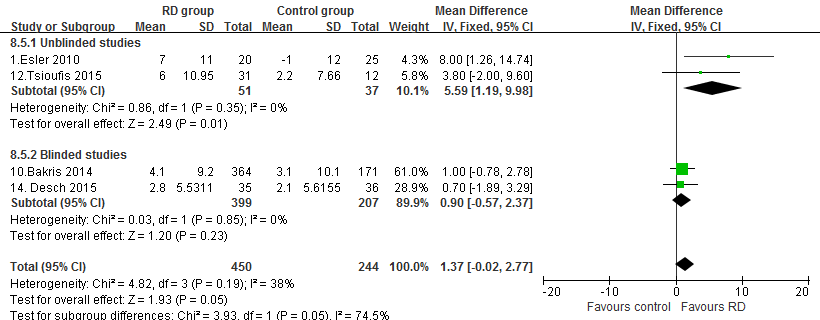


***Supp Fig.3*** Meta-analysis of office-based systolic blood pressure reduction in 6 months in only control arm


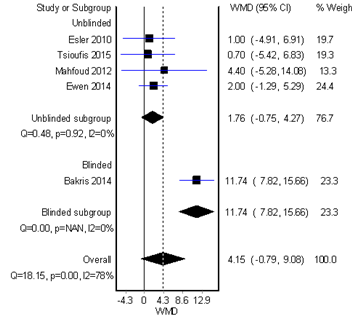


***Supp Fig.4*** Meta-analysis of office-based *diastolic blood pressure* reduction in 6 months in only control arm


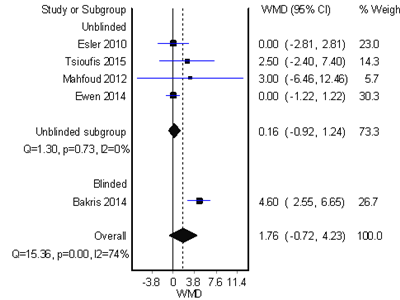


***Supp Fig.5*** Funnel plot. A, blood pressure reduction in 3 months; B & C, systolic blood pressure and *diastolic blood pressure* reduction in 6 months


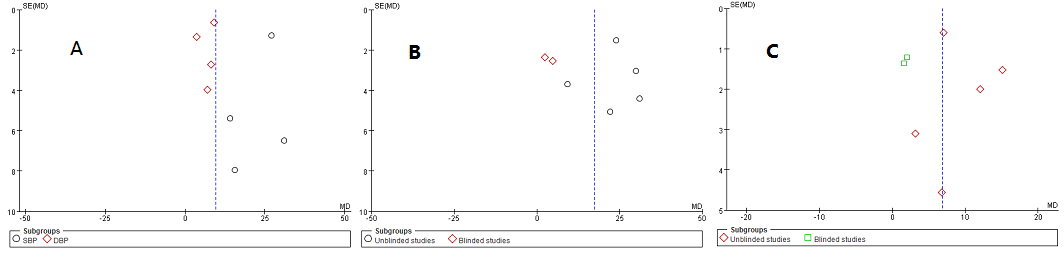

Supplement: Supplemental Digital Content [file medi-95-e3939-s001.doc]
